# The Built Environment, PTSD Symptoms, and Tobacco Use among Permanent Supportive Housing Residents

**DOI:** 10.1007/s10900-024-01422-w

**Published:** 2024-12-16

**Authors:** Mark R. Hawes, Deepalika Chakravarty, Fan Xia, Wendy Max, Margot Kushel, Maya Vijayaraghavan

**Affiliations:** 1 Center for Tobacco Control Research and Education, University of California, San Francisco, CA, USA; 2 UCSF Benioff Homelessness and Housing Initiative, University of California, San Francisco, San Francisco, CA, USA; 3 Center for AIDS Prevention Studies, Division of Prevention Science, University of California, San Francisco, San Francisco, CA, USA; 4 Department of Epidemiology and Biostatistics, University of California, San Francisco, San Francisco, CA, USA; 5 Department of Social and Behavioral Sciences, University of California, San Francisco, San Francisco, CA, USA; 6 Institute for Health & Aging, University of California, San Francisco, San Francisco, CA, USA; 7 Division of General Internal Medicine, San Francisco General Hospital, University of California, San Francisco, San Francisco, CA, USA; 8 Division of Health Equity and Society, University of California, San Francisco, San Francisco, CA, USA; 9 UCSF Division of General Internal Medicine, University of California, San Francisco, San Francisco, CA, USA; 10 530 Parnassus Ave, San Francisco, CA 94143, USA

## Abstract

**Introduction:**

50% of permanent supportive housing (PSH) residents in the U.S. smoke cigarettes, and tobacco-related mortality is their number one cause of death. Over 30% of PSH residents have post-traumatic stress disorder (PTSD), and many perceive their built environment (e.g., housing) as inadequate for mental and physical health recovery. It is unknown whether built environment factors moderate the relationship between PTSD and tobacco use among PSH residents.

**Methods:**

We used baseline data from 400 participants in a smoke-free home intervention in PSH sites in the San Francisco Bay Area between 2022 and 2024. We explored whether perceived housing quality and perceived neighborhood safety moderated the relationship between PTSD symptoms and cigarettes per day (CPD) using linear mixed models.

**Results:**

62.8% of the participants were male, 41.8% were Black, 30.5% screened positive for PTSD, 54.3% rated their housing as average/poor, and the mean neighborhood safety score was 3.4 (SD 0.9). Mean CPD was significantly higher in participants with PTSD compared to those without PTSD among participants who rated their housing as good/excellent (5.1; 95% CI: 2.7, 7.5) or their neighborhood as safer (7.8; 95% CI: 2.8, 12.8). Mean CPD was not significantly different between those with and without PTSD among participants who rated their housing as average/poor or their neighborhood as less safe.

**Conclusions:**

Perceived housing quality and neighborhood safety moderated the association between PTSD symptoms and CPD. Findings have implications for developing trauma-informed, multi-level interventions for tobacco use that combine individually directed approaches with those that consider the built environment.

## Introduction

Approximately 50–60% of the 400,000 people living in permanent supportive housing (PSH) use tobacco [1, 2], and tobacco-related illnesses are responsible for 60% of all-cause mortality in this population [3]. PSH is subsidized housing with supportive services for people with a history of chronic homelessness. PSH is the preferred approach for exiting homelessness for people with disabilities, including mental health and substance use disorders [4]. Housing First is the preferred PSH model, providing low-threshold access to housing without preconditions of abstinence or the requirement to engage in supportive services [5]. PSH provides a stable environment conducive to healing and recovery from trauma, mental health conditions, and substance use [5], which makes PSH an ideal venue to address high rates of tobacco use that frequently co-occur with behavioral health conditions.

Behavioral health conditions are highly prevalent among people living in PSH. For instance, rates of post-traumatic stress disorder (PTSD) are 6 to 12 times higher in PSH residents (30–65%) than in the general population (5%). High rates of PTSD stem from exposure to physical, emotional, or sexual trauma before and during episodes of homelessness [6–10]. In people experiencing homelessness, PTSD is associated with tobacco use to reduce negative affect and stress, and for increased mood and social benefit [6, 11]. The link between PTSD and intensity of tobacco use among PSH residents could have implications for tobacco treatment.

The built environment (i.e., spaces where people live, work, and play) is another factor that may influence tobacco use behaviors [12–14]. Built environments not conducive to overall well-being have been linked to risk behaviors like tobacco [15] and alcohol use [12] and the development of obesity, cancer, and depression [12, 16]. Within PSH, variations in the built environment, like housing quality and perceived neighborhood safety, may influence tobacco use and cessation.

Among people experiencing homelessness, higher scores on the urban life stress scale, which captures components of the built environment, are associated with heavier smoking [15]. Among PSH residents, negative perceptions of housing quality and neighborhood environment increase stress and worsen mental health and quality of life [13], and PSH environments that are not trauma-sensitive (i.e., do not support trauma-informed care principles) increase anxiety, fear, and hypervigilance [17, 18]. Stress and anxiety related to negative built environment factors may increase tobacco use and pose a barrier to cessation [19].

PTSD is closely linked to tobacco use, and several studies have found that adverse neighborhood environments contribute to increased tobacco use [6, 11, 15]. However, it remains unclear whether built environment factors influence the relationship between PTSD and tobacco use among PSH residents. Individuals living in PSH have transitioned from the instability and trauma of homelessness to stable, supportive housing communities, potentially affecting both their PTSD symptoms and tobacco use. Since PSH communities vary widely in housing quality (e.g., infrastructure, services, property management) and neighborhood environments [17, 18], it is crucial to explore how the built environment interacts with trauma and tobacco use to inform the development of interventions that can reduce tobacco-related health inequities among people living in PSH.

Our study sought to address this gap among PSH residents in the San Francisco Bay Area by examining whether screening positive for PTSD was associated with higher cigarette consumption and whether the relationship between a positive PTSD screen and cigarette consumption was moderated by perceived housing quality and perceived neighborhood safety. We hypothesized that poor housing quality and unsafe neighborhoods could impact the relationship between a positive PTSD screen and tobacco use in two possible ways: (a) poor housing quality and less safe neighborhoods could worsen the impact of PTSD on smoking, contributing to a higher consumption among people with PTSD compared to those without PTSD, or (b) poor housing quality and less safe neighborhoods could have a similar effect as PTSD on smoking, contributing to a threshold effect of stressors, with minimal difference in smoking between those screening positive for PTSD and those screening negative.

## Methods

### Participants and Setting

This study used baseline data from a National Cancer Institute (NCI)-funded cluster randomized controlled trial evaluating the efficacy of a multi-level intervention designed to increase the voluntary adoption of smoke-free homes among PSH residents [20, 21]. We conducted the trial in 40 San Francisco Bay Area multi-unit PSH sites and enrolled 400 participants between February 2022 and February 2024. The baseline data from all 400 participants are included in the current analysis. Eligible participants were residents who currently smoked in their homes (smoked at least five cigarettes per day in the past 7 days, verified by expired carbon monoxide, ≥ 8 parts per million) [22], lived at the study sites, were 18 years or older, were English proficient, and able to provide informed consent.

### Measures

#### Cigarette Consumption

The primary outcome was self-reported cigarettes smoked per day (CPD). We asked participants to report whether they smoked in the past 7 days and the number of cigarettes smoked on their smoking days. We used these variables to determine the average CPD [23].

#### Posttraumatic Stress Disorder

The primary independent variable was the Primary Care-PTSD Screen (PC-PTSD-5) [24]. The PC-PTSD-5 screen provides participants with examples of traumatic events (e.g., physical or sexual assault) and asks participants if they have experienced a traumatic event in their lifetime. The screener then asks about PTSD symptoms experienced in the past 30 days. We categorized people who scored 4 and higher as having a positive PTSD screen; we chose this cut-off to reduce false positives [24].

#### Neighborhood Safety

We assessed perceptions of neighborhood safety using the three-item Neighborhood Safety Scale (NSS) [25]. NSS scores can range from 1 to 5, with lower scores representing greater perceptions of neighborhood safety.

#### Housing Quality

We assessed housing quality by asking participants to rate the quality of their current housing. Response options were poor, average, good, and excellent [20]. We dichotomized housing quality as poor or average vs. good or excellent.

#### Sociodemographic Covariates

Self-reported sociodemographic data included age, gender identity (male vs. female), and race/ethnicity (Black/African American, White, Hispanic/Latinx, Asian, Native Hawaiian or other Pacific Islander, American Indian or Alaskan Native, multiracial).

#### Data Analysis

We reported sociodemographic and tobacco use characteristics for all participants using mean and standard deviation for continuous variables and frequencies and proportions for categorical variables. We first examined the association between screening positive for PTSD and cigarette consumption (i.e., CPD) in an unadjusted model. Next, in two separate models, we examined the interaction of (a) housing quality and PTSD and (b) neighborhood safety and PTSD on the outcome of CPD using linear mixed models (LMMs) with a random intercept to address clustering by PSH sites. The analytic sample for our housing quality and neighborhood safety models was 396 and 395, respectively, accounting for missing data on independent variables. We adjusted for age, gender identity, and race/ethnicity in all models. We used Stata 17.0 (Stata Corp LP, College Station, TX, USA) for all analyses.

## Results

### Sample Characteristics

Among the 400 participants, the mean age was 54.5 years (SD 10.7), 32.5% identified as female, and 41.8% as Black or African American (Table 1). Of the participants, 30.5% (*N* = 122) screened positive for PTSD and 54.3% (*N* = 217) rated their housing as average or poor. The NSS score ranged from 1 to 5 with a mean of 3.4 (SD 0.9). Average daily cigarette consumption was 11.1 (SD 7.5).

### Association of PTSD and Cigarette Consumption Moderated by Housing Quality

**Fig. 1:**
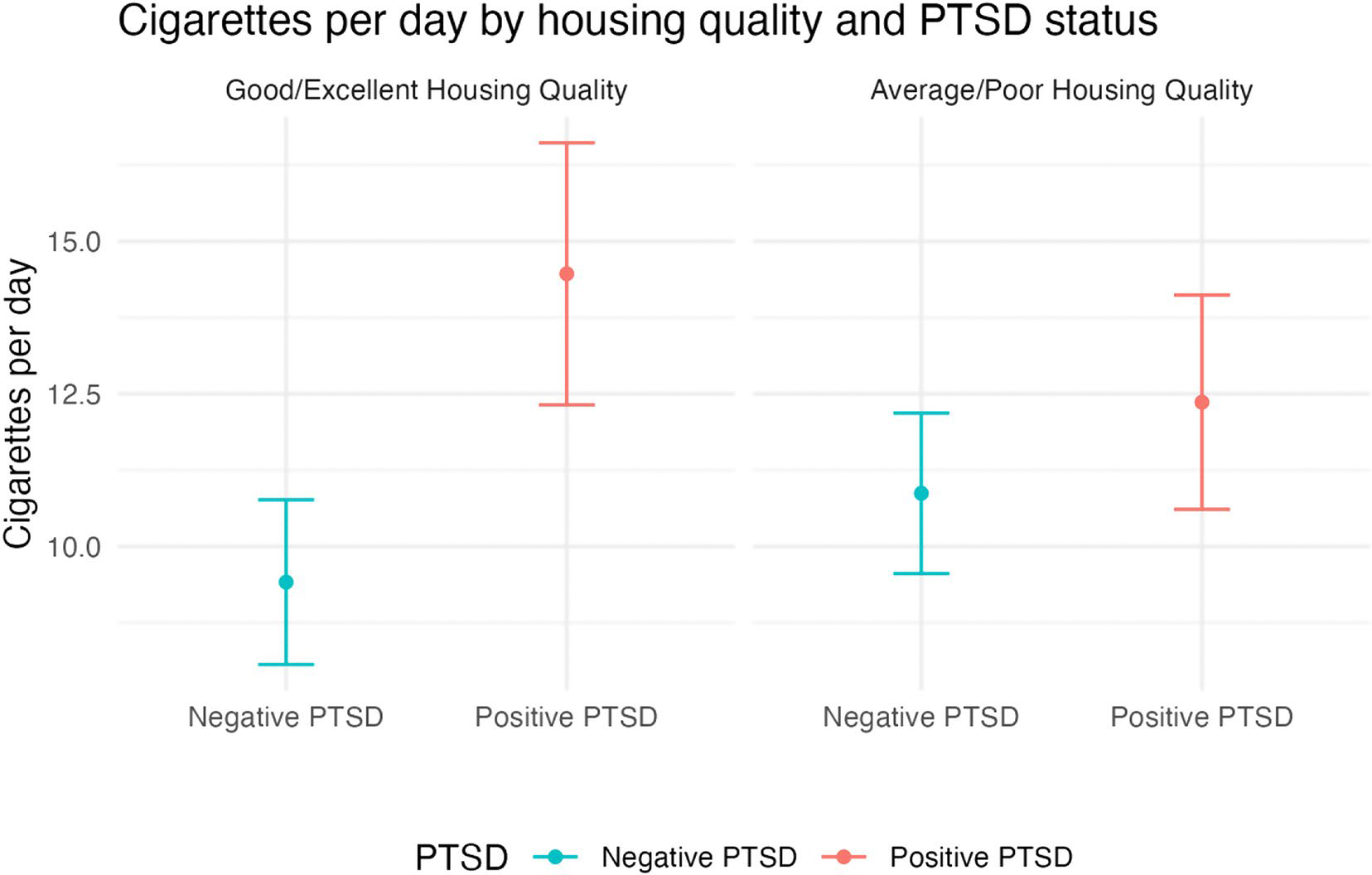
Adjusted^a^ mean cigarettes per day by housing quality^b^ and Post Traumatic Stress Disorder (PTSD)^c^ status (*N* = 396)^d^. Note Confidence intervals (CI) represent tests of statistically significant differences between people with positive and negative PTSD screens. ^a^Regression model adjusted for age, gender, and race/ethnicity. ^b^Housing quality was assessed by asking participants to rate the quality of their current housing. Responses were dichotomized as average or poor vs. good or excellent. ^c^Post Traumatic Stress Disorder (PTSD) was assessed with the Primary Care-PTSD Screen (PC-PTSD-5) (Bovin et al., 2021). A cut point of 4 and above was used to identify PTSD. We chose this cutoff to reduce false positives. ^d^The analytic sample was 396, accounting for missing data on independent variables

In the unadjusted model, people who screened positive for PTSD smoked on average 3.0 more CPD than those screening negative for PTSD (95% CI: 1.47, 4.59). In multivariable analysis, housing quality moderated the relationship between PTSD and cigarette consumption (Supplementary Material). Among participants who rated their housing as good or excellent, the mean CPD was 5.1 (95% CI: 2.65, 7.45) higher in people screening positive for PTSD compared to those who screened negative (Fig. 1). Among those rating their housing as average or poor, the mean CPD difference narrowed to 1.5 between those with and without PTSD (95% CI: −0.56, 3.55) and was not statistically significant (Table 2).

### Association of PTSD and Cigarette Consumption Moderated by Neighborhood Safety

**Fig. 2:**
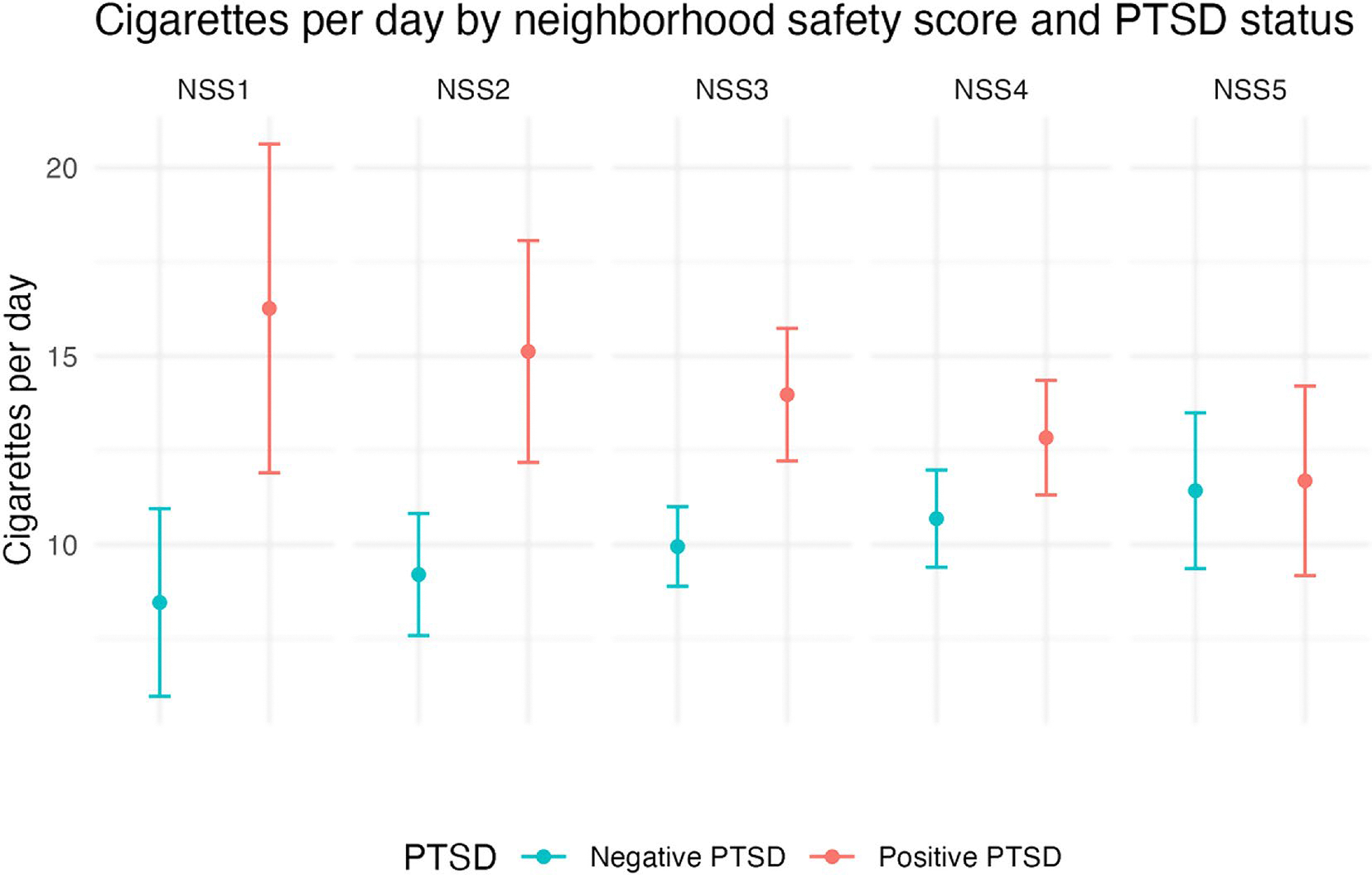
Adjusted^a^ mean cigarettes per day by the Neighborhood Safety Scale (NSS)^b^ score and Post Traumatic Stress Disorder (PTSD)^c^ status (*N* = 395)^d^. *Note* Confidence intervals (CI) represent tests of statistically significant differences between people with positive and negative PTSD screens. ^a^Regression model adjusted for age, gender, and race/ethnicity. ^b^Neighborhood safety was assessed using the Neighborhood Safety Scale (NSS) total score (Mujahid et al., 2007). Scores ranged from 1 to 5; lower neighborhood safety scores indicated more neighborhood safety. ^c^Post Traumatic Stress Disorder (PTSD) was assessed with the Primary Care-PTSD Screen (PC-PTSD-5) (Bovin et al., 2021). A cut point of 4 and above was used to identify PTSD. We chose this cut-off to reduce false positives.^d^The analytic sample was 395, accounting for missing data on independent variables

In the adjusted multivariable analysis, neighborhood safety moderated the relationship between PTSD and cigarette consumption (Supplementary Material). Among participants rating their neighborhood safety as 1 (safest), the mean CPD was 7.8 (95% CI: 2.84, 12.77) higher in people screening positive for PTSD compared to those who screened negative (Fig. 2). Among participants rating their neighborhood safety as 5 (least safe), the mean CPD difference narrowed to 0.3 (95% CI: −2.86 to 3.39) and was not statistically significant (Table 2).

## Discussion

In this study of people living in PSH, we found a significant association between screening positive for PTSD and increased cigarette consumption, consistent with prior literature in the general population and among people experiencing homelessness [26–28]. Our results extend previous research by being the first study to show that perceptions of housing quality and neighborhood safety moderate the relationship between tobacco use and PTSD symptoms.

Among PSH residents who perceived their housing quality as good or excellent, those who screened positive for PTSD smoked significantly more than those who screened negative for PTSD. Similarly, among PSH residents who felt safer in their neighborhood, those screening positive for PTSD smoked significantly more than those who screened negative. Thus, PTSD symptoms may have attenuated the positive effects of living in good-quality housing, highlighting a need to address PTSD symptoms among PSH residents who smoke.

Conversely, when people perceived their housing as inadequate or their neighborhood as less safe, the difference in cigarette consumption between those screening positive for PTSD and those screening negative narrows and is not significantly different. This finding could be due to an overall increase in psychosocial distress among people with poor housing quality and neighborhood environments, which may mitigate the significant differences in smoking found between people with and without PTSD in other studies [6, 11]. Consistent with our second hypothesis, these findings suggest a potential threshold effect of stressors (rather than an additive effect) from PTSD or a negative built environment, such that when housing quality or neighborhood safety are unfavorable, having PTSD does not further increase cigarette smoking.

To assess whether differences in CPD also exist by housing quality and neighborhood safety, we conducted an unadjusted post-hoc sensitivity analysis that compared differences in CPD by housing status and neighborhood environment separately for each PTSD group. Among people screening negative for PTSD, there were no statistically significant differences in cigarette consumption between those who rated their housing quality as good/excellent compared to average/poor (9.4 vs. 10.8, *p* = 0.09) or who rated their neighborhood environment as safer compared to less safe (9.6 vs. 10.9, *p* = 0.13). Among people screening positive for PTSD, there were no statistically significant differences in cigarette consumption between those who rated their housing quality as good/excellent compared to average/poor (14.4 vs. 12.4, *p* = 0.22) or who rated their neighborhood environment as safer compared to less safe (14.2 vs. 12.4, *p* = 0.24). These results suggest that differential tobacco use patterns are based on the synergistic interaction between mental health and built-environment, and highlight a role for multi-level interventions for tobacco use that address the intersection of mental health symptoms and social determinants of health like housing and neighborhood environment.

Among participants in our study, 83% reported a traumatic event in their lifetime, and 30.5% screened positive for PTSD. There is a bidirectional link between tobacco use and mental health [29, 30]. Individuals may use tobacco to self-medicate, cope with anxiety or stress from trauma, or treat PTSD symptoms, which, in turn, may exacerbate mental health symptoms [29, 31]. Tobacco cessation may help alleviate mental health symptoms, and treating mental health concerns can help support tobacco cessation [29]. Our findings underscore the importance of addressing PTSD symptoms when discussing tobacco cessation and emphasizing the potential for stress and anxiety to improve with tobacco cessation [32]. A key component of tobacco treatment in PSH might include recognizing signs of trauma and its connection with tobacco use and providing trauma-informed cessation care, including trauma-sensitive housing [33, 34].

Providing access to trauma-sensitive housing and safe neighborhoods is aligned with recommendations for trauma-informed tobacco cessation treatment [33, 34]. Among women living in single-room occupancy hotels, trauma-insensitive housing (e.g., street-level violence, chaotic housing spaces, pest infestations, shared bathrooms, in-operable door locks) negatively affects behavioral health [17]. Conversely, trauma-sensitive housing environments can increase housing stability and improve mental health [17, 18]. PSH that follows trauma-informed care principles may help rebuild a sense of empowerment by providing safety, ensuring privacy, reducing adverse environmental stimuli, providing space for healthy physical boundaries, and offering opportunities for peer-led support and skill-building groups [17, 18, 35]. Training PSH staff in the 4 Rs of trauma-informed care (Realize, Recognize, Respond, and Resist) will provide a key component of trauma-sensitive environments [36], which may support tobacco cessation among those with PTSD.

While improving PSH housing quality and neighborhood conditions may require increased funding for infrastructure and strategic planning, our findings suggest that PSH organizations can take immediate steps to improve their housing environment to support tobacco cessation. Creating opportunities for residents to make positive connections with staff and neighbors through community events and life skills classes can improve well-being and quality of life [13]. Moreover, ensuring consistent access to onsite physical and mental health care is vital to support healing and recovery after periods of homelessness and is consistent with the Housing First approach of PSH [37]. By addressing these factors, PSH communities can play a pivotal role in reducing smoking rates and promoting long-term cessation among residents.

### Limitations

Our study has some limitations. The use of self-reported data introduces the possibility of reporting bias. The cross-sectional nature of this analysis precludes a causal interpretation of the findings. Our sample of PSH residents who smoke cigarettes was drawn from 40 multi-unit, project-based PSH sites in the San Francisco Bay area, which may limit generalizability to other parts of California or the US. However, this PSH model is similar in structure to PSH in other large cities in the Western United States, where a large portion of people experiencing homelessness live.

## Conclusion

This study highlights the interplay between the built environment, trauma, and tobacco use among people living in PSH. Our findings underscore the need to create trauma-sensitive housing and neighborhood environments and provide trauma-informed tobacco cessation treatment to reduce tobacco use among PSH residents. This can occur through increased community-building events that provide social support and foster connections, ensuring onsite access to mental and physical health, and training staff in the principles of trauma-informed care. Creating stable housing environments and communities that support mental health recovery and well-being will increase tobacco cessation and reduce the high burden of tobacco-related health inequities among PSH residents.

## Supplementary Material

Supplemental Tables

## Figures and Tables

**Table 1:** Baseline sample and clinical characteristics among people living in permanent supportive housing and participating in the smoke- free home study (*N* = 400)

**Sociodemographics**	
Age (Mean, SD)	54.5 (10.7)
Gender^a^ (N, %)	
Male	251(62.8)
Female	130 (32.5)
Transgender or non-binary	17 (4.3)
Race/ethnicity^a^ (N, %)	
Black or African American	167 (41.8)
White	100 (25.0)
More than one race	48 (12.0)
Hispanic/Latino	62 (15.5)
American Indian/Alaska Native	10 (2.5)
Asian	4 (1.0)
Native Hawaiian or other Pacific Islander	4 (1.0)
**Post Traumatic Stress Disorder (PTSD)** ^b^ **(Mean, SD)**	
Positive PC-PTSD-5 screen	122 (30.5)
**Built environment moderators**	
Housing quality^c^ (N, %)	
Good-Excellent	183 (45.8)
Average-Poor	217 (54.3)
Neighborhood safety scale (NSS) total score^d^ (Mean, SD)	3.4 (0.9)
**Tobacco use**	
Cigarettes smoked per day (Mean, SD)	11.1 (7.5)
First cigarette within 5 min of waking (N, %)	156 (39.0)

*Note*

a Two participants did not report their gender, and five participants did not report their race/ethnicity, resulting in percentages that do not add up to 100%

b Post Traumatic Stress Disorder (PTSD) was assessed with the Primary Care-PTSD Screen (PC-PTSD-5) (Bovin et al., 2021). A cut point of 4 and above was used to identify PTSD. We chose this cut-off to reduce false positives

c Housing quality was assessed by asking participants to rate the quality of their current housing. Responses were dichotomized as average or poor vs. good or excellent

d Neighborhood safety was assessed using the Neighborhood Safety Scale total score (Mujahid et al., 2007). Scores ranged from 1 to 5; lower neighborhood safety scores indicate more neighborhood safety

**Table 2:** Adjusted^a^ differences in mean cigarettes per day (CPD) between those with and without Post-traumatic stress disorder (PTSD) by perceived housing quality (*N* = 396)^d^ and the Neighborhood Safety Scale score (*N* = 395)^d^ (NSS)

	Difference in mean CPD^a^ between those with and without PTSD^b^	95% CI	

Housing quality			
Good/excellent housing quality	5.1	**2.65**	**7.45**
Average/poor housing quality	1.5	−0.56	3.55
Neighborhood Safety Scale score (NSS)^c^			
NSS = 1	7.8	**2.84**	**12.77**
NSS = 2	5.9	**2.64**	**9.20**
NSS = 3	4.0	**2.13**	**5.94**
NSS = 4	2.2	**0.33**	**3.97**
NSS = 5	0.3	−2.86	3.39

*Note*

a CPD represents the adjusted differences in mean cigarettes smoked per day and is based on the mixed effects regression model, adjusted for age, gender, and race/ethnicity

b Post Traumatic Stress Disorder (PTSD) was assessed with the Primary Care-PTSD Screen (PC-PTSD-5) (Bovin et al., 2021). A cut point of 4 and above was used to identify PTSD. We chose this cut-off to reduce false positives

c Neighborhood safety was assessed using the Neighborhood Safety Scale total score (Mujahid et al., 2007). Scores range from 1 to 5; lower neighborhood safety scores indicate more neighborhood safety

d The analytic sample for our housing quality and neighborhood safety models was 396 and 395, respectively, accounting for missing data on independent variables

## Data Availability

Deidentified data are available upon request.
