# Supplementary material for: The Built Environment, PTSD Symptoms, and Tobacco Use among Permanent Supportive Housing Residents
Source: J Community Health. Author manuscript; Available in PMC 2025 Apr 1. (PMC11936717; doi:10.1007/s10900-024-01422-w)
Supplement: Supplementary file 1 [file NIHMS2059385-supplement-Supplemental_Tables.docx]

**Supplementary Materials Table 1.** Association of Post Traumatic Stress Disorder (PTSD) and cigarette consumption moderated by housing quality (N=396)^d^

| Outcome: mean cigarettes per day | Coef | SE | 95% CI |  |
| --- | --- | --- | --- | --- |
| Age | 0.01 | 0.04 | -0.06 | 0.08 |
| Gender (ref: male) |  |  |  |  |
| Female | -1.18 | 0.79 | -2.72 | 0.36 |
| Transgender or non-binary | 0.81 | 1.81 | -2.73 | 4.34 |
| Race/ethnicity (ref: white) |  |  |  |  |
| Black | -1.91 | 0.94 | -3.76 | -0.06 |
| Hispanic | -1.41 | 1.17 | -3.70 | 0.87 |
| All other races^a^ | -0.87 | 1.12 | -3.06 | 1.32 |
| Housing quality^b^ (ref: good/excellent) |  |  |  |  |
| average/poor | 1.46 | 0.86 | -0.24 | 3.15 |
| PTSD^c^ (ref: negative screen) |  |  |  |  |
| positive screen | 5.05 | 1.22 | **2.65** | **7.45** |
| PTSD (ref: negative screen)*Housing quality (ref: good/excellent) |  |  |  |  |
| average/poor*positive screen | -3.56 | 1.58 | **-6.66** | **-0.46** |
|  |  |  |  |  |
| Model P-value | p<0.001 |  |  |  |

Note

^a^Other races included Asian, American Indian, Alaska Native, Native Hawaiian, other Pacific Islander, multi/biracial, and participants who did not report their race.

^b^Housing quality was dichotomized as average or poor vs. good or excellent.

^c ^Post Traumatic Stress Disorder (PTSD) was assessed with the Primary Care-PTSD Screen (PC-PTSD-5) (Bovin et al., 2021). A cut point of 4 and above was used to identify PTSD. We chose this cut-off to reduce false positives.

Cigarette consumption is the mean number of cigarettes smoked per day.

^d^The analytic sample was 396, accounting for missing data on independent variables.

**Supplementary Materials Table 2.** Association of Post Traumatic Stress Disorder (PTSD) on cigarette consumption moderated by the Neighborhood Safety Scale (NSS) score (N=395)^d^

| Outcome: mean cigarettes per day | Coef | SE | 95% CI |  |
| --- | --- | --- | --- | --- |
| Age | 0.01 | 0.04 | -0.07 | 0.08 |
| Gender (ref: male) |  |  |  |  |
| Female | -1.33 | 0.79 | -2.88 | 0.22 |
| Transgender or non-binary | 0.73 | 1.81 | -2.81 | 4.28 |
| Race/ethnicity (ref: White) |  |  |  |  |
| Black | -1.73 | 0.95 | -3.58 | 0.13 |
| Hispanic | -1.43 | 1.17 | -3.72 | 0.86 |
| All other races^a^ | -0.87 | 1.13 | -3.08 | 1.34 |
| Neighborhood safety score (NSS)^b^ | 0.74 | 0.52 | -0.27 | 1.76 |
| PTSD^c^ (ref: negative screen) |  |  |  |  |
| positive screen | 9.69 | 3.43 | **2.96** | **16.42** |
| PTSD (ref: negative screen)*NSS |  |  |  |  |
| positive screen | -1.89 | 0.94 | **-3.73** | **-0.04** |
| Model P-value | P=0.001 |  |  |  |

Note

^a^Other races included Asian, American Indian, Alaska Native, Native Hawaiian, other Pacific Islander, multi/biracial, and participants who did not report their race.

^b^Neighborhood safety was assessed using the Neighborhood Safety Scale (NSS) total score (Mujahid et al., 2007). Scores ranged from 1 to 5; lower neighborhood safety scores indicate more neighborhood safety.

^c ^Post Traumatic Stress Disorder (PTSD) was assessed with the Primary Care-PTSD Screen (PC-PTSD-5) (Bovin et al., 2021). A cut point of 4 and above was used to identify PTSD. We chose this cut-off to reduce false positives.

Cigarette consumption is the mean number of cigarettes smoked per day.

^d^The analytic sample was 395, accounting for missing data on independent variables.
